# Parental Monitoring, Individual Dispositions, and Alcohol Use Disorder: A Longitudinal Study with Young Swiss Men

**DOI:** 10.3390/ijerph18189938

**Published:** 2021-09-21

**Authors:** Jimena Sobrino-Piazza, Simon Foster, Natalia Estévez-Lamorte, Meichun Mohler-Kuo

**Affiliations:** 1 La Source, School of Nursing Sciences, HES-SO University of Applied Sciences and Arts of Western Switzerland, 1004 Lausanne, Switzerland; j.sobrinopiazza@ecolelasource.ch; 2 Department of Child and Adolescent Psychiatry and Psychotherapy, University Hospital of Psychiatry Zurich, University of Zurich, 8032 Zurich, Switzerland; simon.foster@kjpd.uzh.ch (S.F.); natalia.estevezgomez@uzh.ch (N.E.-L.)

**Keywords:** alcohol use disorder, parental monitoring, sensation seeking, coping strategies, interaction effects

## Abstract

This paper evaluates the interaction between level of parental monitoring in adolescence and individual dispositions present in early adulthood in the prediction of alcohol use disorder (AUD) in the mid-20s. Data were drawn from the Cohort Study on Substance Use Risk Factors (C-SURF), encompassing 4844 young Swiss men who were surveyed three times within a 5-year period. The outcome variable was alcohol use disorder (AUD) as defined in the DSM-5. Independent variables were sensation seeking (Brief Sensation Seeking Scale) and the coping strategies active coping and denial (Brief COPE). Low parental monitoring, high sensation seeking, and high denial were found risk factors of AUD (odds ratio (OR) = 1.21 (1.05–1.40); OR = 1.56 (1.37–1.78); OR = 1.15 (1.01–1.31)). A significant interaction effect was identified between active coping and parental monitoring; high active coping in early adulthood was found protective of AUD, only among individuals who had low parental monitoring in adolescence (OR = 0.70 (0.52–0.96)). In addition to interventions to upskill parents for improving monitoring, other interventions directed to young adults who had disadvantaged family contexts could be implemented, with the aim of enhancing the use of adaptive coping strategies such as active coping. Prevention targeting avoidant coping strategies and sensation seeking should be privileged too.

## 1. Introduction

Alcohol use disorder (AUD) is one of the most prevalent mental disorders in the Western world [1,2]. It is highly disabling and has devastating health consequences that include various comorbidities [3] and premature death [4].

Individuals who initiate alcohol consumption at an early age have an increased risk of AUD [5,6]. In this regard, there is extensive evidence examining the role that parents might play in their offspring’s alcohol initiation and alcohol use/misuse during adolescence and early adulthood [7,8,9]. Parental monitoring (i.e., the extent to which parents know about their children’s whereabouts and the company they spend time with) has been identified as the strongest protective parental factor against alcohol use/misuse [7]. The corollary to this is that the absence of adequate parental monitoring during childhood and adolescence may be a form of neglect—in the sense of the WHO’s definition of “emotional neglect”, which is “failure of a parent to provide for the emotional development of the child—where the parent is in a position to do so” [10]. As such, it may be a risk factor for alcohol use/misuse [11]. Evidence from longitudinal studies suggests that the effects of parenting factors (parent–child relationship, favorable attitudes towards alcohol use, family conflict, parental support, parental involvement) persist over time, even though their impact may fade [7].

Previous studies have also identified certain individual dispositions that are associated with alcohol use/misuse, including personality traits and coping strategies. Among these personality traits, sensation seeking (i.e., “the need for varied, novel, and complex sensations and experiences and the willingness to take physical and social risks for the sake of such experiences” [12]) has been found to predict risky drinking patterns [13,14]. With respect to coping, the use of avoidant coping strategies also appears to be directly associated with alcohol use [15,16]. Individuals with AUD have been documented to use more avoidant coping and fewer problem-focused coping strategies than controls [17]. Evidence has also been uncovered that healthy, problem-focused coping strategies protect against relapse among individuals in recovery from AUD [18].

The overall purpose of the current paper is to shed light on how parenting factors and individual dispositions interact as predictors of AUD in early adulthood. More specifically, our research evaluated whether a disadvantaged family context in adolescence—characterized by below-average parental monitoring—affects all individuals in their mid-20s equally as a predictor of AUD, or if specific individual dispositions present in early adulthood (e.g., level of sensation seeking and use of avoidant or problem-focused coping strategies) either intensify or buffer against this effect over time. To the best of our knowledge, no previously published studies have explored potential interactions between level of parental monitoring and individual dispositions longitudinally, analyzing how various combinations of these potential risk and protective factors for AUD might relate over time, during a person’s transition from adolescence to their mid-20s.

## 2. Materials and Methods

### 2.1. Study Design

For our analysis, longitudinal data from the Cohort Study on Substance Use Risk Factors (C-SURF)—a prospective cohort study that has followed a representative sample of young Swiss adult men over 10 years—were used. For C-SURF, participants were recruited through the military recruitment system. In Switzerland, at the approximate age of 19 years, all Swiss men are summoned to an army recruitment center to determine if they are eligible for military service, civil service, or neither. Since conscription is mandatory, and since there are no preselection criteria prior to it, Swiss army recruitment centers provide access to virtually the entire Swiss male population at age 19. Between August 2010 and November 2011, young men who presented at three of the six Swiss army recruitment centers were asked to participate in C-SURF. These three recruitment centers cover 21 of the 26 cantons in Switzerland, including all the French-speaking and a majority of the German-speaking regions. The sample is thus representative of most cantons and reflects the two main languages in the country, as well as both rural and urban regions. To enroll in the C-SURF study, participants were required to give their informed written consent. The military facilities were exclusively used for the enrollment process. All individuals who consented to participate either received an email containing a link to the study’s online questionnaire or, if they requested it, a printed version sent to their home address. A first follow-up questionnaire was sent out 15 months later (2012–2014), followed by a second follow-up questionnaire approximately 65 months after enrollment (2016–2018) and a third follow-up questionnaire more recently (2019–2020). Each questionnaire required roughly 45–60 min to complete, with remuneration provided for questionnaire completion in the form of monetary vouchers, ranging in value from 30 to 60 Swiss francs, depending on the data collection wave. All data were anonymized. 

Prior to any data collection, the study protocol was approved by Lausanne University Medical School’s Ethics Committee for Clinical Research (Protocol No. 15/07). The present study uses data from C-SURF’s baseline assessment and from its first and second follow-up assessments (FU1 and FU2).

### 2.2. Participants

Out of the 7556 conscripts who signed up to participate in the study, 5987 (79.2%) participated in the baseline survey. Of these, 5479 also completed the first follow-up survey FU1 (retention rate: 91.5%); out of these, 4981 also completed the second follow-up questionnaire FU2 (retention rate with respect to baseline: 83.2%). Sampling procedures and potential nonresponse bias have been described elsewhere [19,20]. The current analysis was restricted to individuals who participated in all three surveys—baseline, FU1, and FU2. Due to missing data for the variables of interest, 137 men were excluded from analysis, leaving a net sample for the present study of 4844 individuals (80.9% of the initial 5987 cases).

### 2.3. Measures

#### 2.3.1. Alcohol Use Disorder (AUD)

Alcohol use disorder (AUD) was measured as defined in the DSM-5, based on 11 alcohol use disorder criteria experienced over the preceding 12 months. The variable was dichotomized so that it was coded as 1, meaning “AUD”, if two or more criteria were met and as 0, meaning “no AUD”, otherwise [21,22]. The present study used measures of AUD from two different points of time, with AUD at FU2 being the outcome variable, and AUD at baseline being a potential confounder for use during analysis. 

#### 2.3.2. Parental Monitoring

Level of parental monitoring was measured at baseline using two questions from the European School Survey Project on Alcohol and Other Drugs (ESPAD), both asking respondents about their situation when they were 15 years old. The two items selected were: “My parents knew where I spent my evenings” and “My parents knew with whom I spent my evenings”. Respondents answered using a 5-point Likert scale ranging from 1 “Almost always” to 5 “Almost never”. The two items’ scores then were averaged. Mean scores up to a value of 2 (“Often”)—the median value of the scale for all respondents at baseline—were coded as 0, meaning “median/high-level parental monitoring”, while scores higher than 2 were coded as 1, meaning “low-level parental monitoring”.

#### 2.3.3. Coping Strategies

Two coping strategies were analyzed in the present study: active coping and denial. Coping strategies were measured at first follow-up (FU1) using the Brief COPE questionnaire ([23], employing both a French version [24] and German version, [25]). This instrument asks participants to evaluate—using a 4-point scale ranging from 1 (“I usually don’t do this at all”) to 4 (“I usually do this a lot”)—how often they do certain things when confronted with a difficult or stressful situation. Each coping strategy was assessed through two items, with these two scores summed to generate scale scores ranging from 2 to 8. The scales then were dichotomized, centered around their median value for all respondents at FU1. As such, scores equal to or below the median were coded as 0, meaning “median/low” use of the coping strategy, while scores above the median were coded as 1, meaning “high” use of the coping strategy.

#### 2.3.4. Sensation Seeking

Sensation seeking was measured at baseline using the Brief Sensation Seeking Scale [26]. This instrument asks respondents to evaluate—using a 5-point scale ranging from 1 “strongly disagree” to 5 “strongly agree”—eight statements assessing their need for novel, exciting, and unpredictable experiences. Scale scores were computed by averaging the items. The scale then was dichotomized around the median score for all respondents of the baseline questionnaire (3.125), with scores equal to or below the median coded as 0, meaning “median/low sensation seeking”, while scores higher than the median were coded as 1, meaning “high sensation seeking”.

#### 2.3.5. Confounding Variables

Sociodemographic variables included in the analyses were financial autonomy and civil status at FU2. The first was coded as one of three categories: 0 “financially autonomous”, 1 “partially financially dependent”, and 2 “financially dependent”. The second was dichotomized into 0 “single” or 1 “living with a partner/married”.

### 2.4. Statistical Analysis

Logistic regression analysis was conducted to explain AUD at FU2, testing a total of four models. The first model, Model 0, examined all potential predictors of AUD—parental monitoring, active coping, denial, and sensation seeking, controlling also for AUD at baseline, financial autonomy, and civil status. Models 1 to 3 each added a different interaction effect to Model 0: Model 1 assessed the interaction between parental monitoring and active coping, Model 2 assessed the interaction between parental monitoring and denial, and Model 3 assessed the interaction between parental monitoring and sensation seeking. We further calculated the predicted probabilities of AUD for those significant interaction effects, across combinations of different levels of parental monitoring and the interacting variable, while all other predictors were fixed as their typical value, as outlined by Fox [27]. All analyses were conducted utilizing R statistical software [28]. Regression analysis was performed employing the glm-function in the base package, while the effect-function from the add-on package “effects” was used to investigate interaction effects [27].

## 3. Results

Characteristics of the sample are summarized in Table 1. The mean age of the 4844 participants at FU2 was 25.4 years (SD = 1.2, range = 23–33), and the prevalence of AUD at FU2 was 31.9%.

### 3.1. Main Effects

Results of the regression analyses are summarized in Table 2. Model 0 revealed statistical evidence that low-level parental monitoring, high-level denial, and high-level sensation seeking all were significant risk factors for AUD (OR = 1.21, 95% CI = 1.05–1.40, *p*-value = 0.011, for low parental monitoring; OR = 1.15, 95% CI = 1.01–1.31, *p*-value = 0.039, for high denial; OR = 1.56, 95% CI = 1.37–1.78, *p*-value <0.0001, for high sensation seeking). No statistical evidence was found of any association between active coping and AUD.

### 3.2. Interaction Effects

Model 1 revealed, however, an interaction effect between active coping and parental monitoring (Table 2). High active coping was found to be protective against AUD when combined with low parental monitoring (OR = 0.70, 95% CI = 0.52–0.96, *p*-value = 0.025). No statistical evidence was found of interactions between either denial or sensation seeking and parental monitoring (Models 2 and 3, respectively).

**Figure 1:**
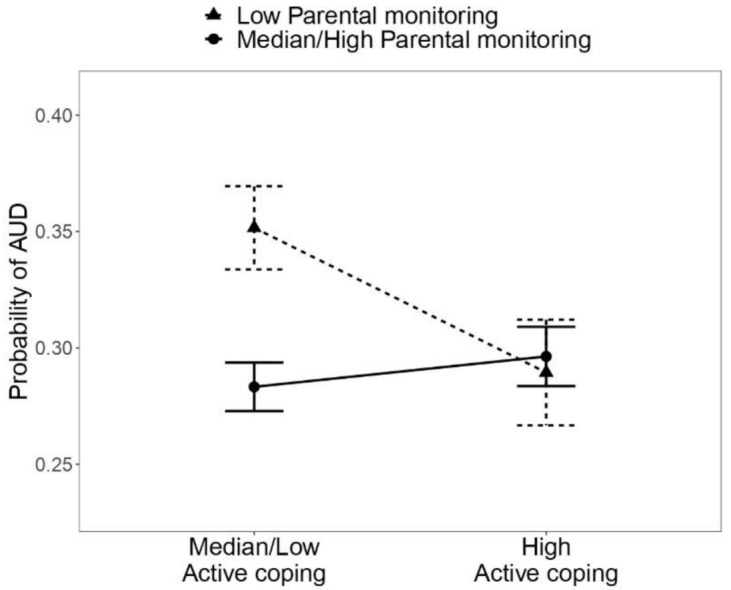
Probabilities of alcohol use disorder predicted by the logistic regression (Model 1) across different levels of parental monitoring and active coping.

The interaction effect between active coping and parental monitoring is depicted in Figure 1 and Table 3. For individuals with adequate levels of parental monitoring, the risk of AUD remained stable, independent of the level of active coping, with predicted probabilities of AUD ranging from 0.28 to 0.30 (SE = 0.01). Among individuals with low parental monitoring, the risk of AUD was moderated by the level of active coping. The combination of low parental monitoring and median/low active coping was associated with an increased risk of AUD, the predicted probability of AUD being 0.35 (SE = 0.02). High levels of active coping, on the other hand, buffered against the risk conveyed by low parental monitoring, as evidenced by a reduction in the estimated probability of AUD to 0.29 (SE = 0.02), similar to the risk of AUD observed among subjects reporting adequate levels of parental monitoring.

## 4. Discussion

The present study examined relationships between the level of parental monitoring during adolescence and individual dispositions present in early adulthood—active coping and denial as coping strategies and sensation seeking as a personality trait—as predictors of alcohol use disorder (AUD) among young Swiss males in their mid-20s. Evidence was found that low levels of parental monitoring during adolescence increased the risk of AUD in these mid-20s young men. This is consistent with previous longitudinal studies that have identified long-lasting effects of parenting factors on alcohol use and misuse [7].

With respect to the individual dispositions, both denial and sensation seeking were found to be risk factors for AUD, which again corresponds with findings from previous research, both regarding the association between high levels of sensation seeking and risky drinking behavior [13,14] and with respect to the direct association between the use of avoidant coping strategies and alcohol use [15,16].

Our study also revealed evidence suggesting that active coping in early adulthood can buffer against the effect of low parental monitoring on AUD. Indeed, active coping in early adulthood was only found to be a protective factor against AUD among those reporting low-level parental monitoring in the past. Returning to the initial question guiding this study, disadvantaged family context marked by low parental monitoring would, thus, not affect all individuals equally as a predictor of AUD among men in their mid-20s. Indeed, those with high active coping strategies seem less affected by this parenting factor and appear to have a level of AUD risk in their mid-20s that is comparable to those who have had adequate parental monitoring during their adolescence.

It is worth comparing the findings we obtained pertaining to sensation seeking and denial—both risk factors of AUD that work independently from parental monitoring—with respect to the effect identified among active copers, where they seem protective against AUD, but only when present in combination with low-level parental monitoring. These results echo what Aldao and Nolen-Hoeksema found when they studied emotion regulation strategies [29]. Using the Brief COPE scale, these investigators identified adaptive strategies (positive reframing and acceptance) as protective against psychopathology symptoms, but only when the levels of maladaptive strategies (rumination, suppression, behavioral disengagement, and denial) were elevated. On the other hand, they identified maladaptive strategies as risk factors for psychopathology symptoms, without identifying any interaction. These results coincide with ours, in that adaptive strategies only appear to make a difference and act as protective under adverse conditions.

Even if the effects found in this paper are not big—odds ratios below 1.5 to 1 are generally considered as small effects [30]—these findings have important implications for the way in which interventions intended to prevent alcohol misuse can be conceived. Prevention should aim to address the risk factors of low parental monitoring, high sensation seeking, and high use of avoidant coping strategies. With respect to individual dispositions, targeted interventions for sensation seekers and users of avoidant coping strategies should be emphasized. In this sense, there is evidence indicating that personality-targeted interventions perform better than traditional substance use interventions [31]. In the case of parental monitoring, prevention may start through interventions to upskill parents in their parental roles and, thereby, prevent the childhood and adolescent alcohol misuse that can stem from inadequate parental monitoring. Our findings reinforce the need for such preventive approaches by illuminating the long-lasting effect that inadequate parental monitoring has, persisting at least until some individuals reach their mid-20s. However, our finding that active coping can act as a protective factor against AUD in adverse conditions indicates that some youths are resilient against AUD, even under adverse early-life conditions.

In addition to interventions designed to help parents augment their parenting skills, other interventions directed towards high-risk young adults could be implemented, with the aim of enhancing the use of adaptive coping strategies like active coping among those belonging to families that otherwise place them at increased risk for AUD. A person’s third decade of life (ages 20–29) is generally considered a window of time during which positive changes in drinking behaviors remain possible [32] but after which substantial changes in drinking behavior become more difficult [33]. More research is needed to determine if active coping strategies can buffer against the effects of other childhood and adolescent risk factors associated with future alcohol misuse.

## 5. Strengths and Limitations

The present study used a large representative cohort sample of young men who were surveyed three times within a 5-year period. A dataset including certain characteristics of extreme interest to us allowed us to analyze for interaction effects longitudinally in men between the ages of 20 and 25, a period typically marked by multiple changes as individuals transition between adolescence and adulthood. However, the study also was subject to several limitations. First, the data were from young Swiss men only, rendering it vital for future studies to evaluate whether the results we observed can be generalized to other populations. For example, potential cultural differences could exist in parental monitoring, both in terms of its definition and its influence on alcohol misuse, as previous research has found [34]. A second study limitation is that our analysis was restricted to data collected on those who agreed to participate in all three consecutive waves of the survey, creating the potential for significant selection bias. However, we ran some additional analyses to identify potential differences between the individuals who were included in our analysis and those who were excluded from it, because of not having answered all three waves, and no significant differences were found in terms of the prevalence of AUD between the two groups, neither at baseline nor at FU2. A third study limitation is that the variable “parental monitoring at age 15” was measured retrospectively among respondents when they averaged 20 years old, and these results might be subject to recall bias. Finally, our measurements relied on self-reports, which may introduce the risk of social desirability bias. On the other hand, our variables were collected using well-validated self-report instruments, and previous research assessing self-report measures of alcohol consumption has demonstrated their validity [35,36].

## 6. Conclusions

In conclusion, we found that low-level parental monitoring during adolescence continues to be a risk factor for AUD among young men into their mid-20s. High sensation seeking and the use of denial as a coping strategy during early adulthood are two more risk factors for AUD. Conversely, active coping during early adulthood appears to be protective against AUD, albeit only among those with inadequate parental monitoring. Preventive interventions should strive to address the above-mentioned three risk factors for AUD and include interventions intended to enhance the protective practice of active coping.

## Figures and Tables

**Table 1:** Sample composition.

	n (%)/ Mean ± SD (Range)	AUD ^a^ (Prevalence in %)	χ^2^ (df) ^d^	*p*-Value
Total	4844	31.9		
Age ^a^	25.4 ± 1.2 (23–33)			
Parental monitoring ^b^				
Low	1248 (25.8)	38.3	31.3 (1)	<0.0001
Median/High	3596 (74.2)	29.7		
Active coping ^c^				
Median/Low	2944 (60.8)	32.5	1.3 (1)	0.261
High	1900 (39.2)	31.0		
Denial ^c^				
Median/Low	2924 (60.4)	30.1	11.9 (1)	0.0006
High	1920 (39.6)	34.8		
Sensation seeking ^b^				
Median/Low	2607 (53.8)	25.3	115.1 (1)	<0.0001
High	2237 (46.2)	39.7		
AUD 5 years earlier ^b^				
No	3333 (68.8)	21.3	552.8 (1)	<0.0001
Yes	1511 (31.2)	55.3		
Level of financial autonomy ^a^				
Financially autonomous	2832 (58.5)	28.2	45.0 (2)	<0.0001
Partially financially dependent	1476 (30.5)	37.9		
Financially dependent	536 (11.1)	35.1		
Civil status ^a^				
Single	4046 (83.5)	33.3	22.3 (1)	<0.0001
Living with a partner/married	798 (16.5)	24.8		

SD = standard deviation; AUD = alcohol use disorder (DSM–5); ^a^ measurement from FU2; ^b^ measurement from baseline; ^c^ measurement from FU1; ^d^ Pearson’s chi-squared test statistic for contingency tables with degrees of freedom in brackets.

**Table 2:** Results of logistic regressions predicting alcohol use disorder.

	AUD ^c^											
	Model 0			Model 1			Model 2			Model 3		
	OR	95% CI	*p*-Value	OR	95% CI	*p*-Value	OR	95% CI	*p*-Value	OR	95% CI	*p*-Value
Low parental monitoring ^a^	1.21	1.05–1.40	0.011	1.37	1.14–1.65	0.001	1.32	1.09–1.60	0.004	1.27	1.02–1.59	0.033
High active coping ^b^	0.97	0.85–1.11	0.682	1.07	0.91–1.25	0.426	0.97	0.85–1.11	0.679	0.97	0.85–1.11	0.685
Low parental monitoring ^a^ × High active coping ^b^				0.70	0.52–0.96	0.025						
High denial ^b^	1.15	1.01–1.31	0.039	1.15	1.01–1.31	0.040	1.22	1.04–1.43	0.013	1.15	1.01–1.31	0.039
Low parental monitoring ^a^ × High denial ^b^							0.81	0.61–1.09	0.167			
High sensation seeking ^a^	1.56	1.37–1.78	<0.0001	1.56	1.37–1.78	<0.0001	1.56	1.37–1.78	<0.0001	1.60	1.37–1.86	<0.0001
Low parental monitoring ^a^ × High sensation seeking ^a^										0.92	0.68–1.23	0.558
AUD 5 years earlier ^a^	4.13	3.61–4.72	<0.0001	4.12	3.60–4.72	<0.0001	4.12	3.60–4.72	<0.0001	4.13	3.61–4.73	<0.0001
Partially financially dependent ^c,d^	1.54	1.33–1.77	<0.0001	1.54	1.33–1.78	<0.0001	1.54	1.33–1.78	<0.0001	1.54	1.33–1.77	<0.0001
Financially dependent ^c,d^	1.41	1.14–1.74	0.002	1.40	1.13–1.73	0.002	1.41	1.14–1.74	0.001	1.41	1.14–1.74	0.002
Living with a partner/married ^c,e^	0.75	0.62–0.90	0.003	0.75	0.62–0.90	0.002	0.75	0.62–0.90	0.003	0.75	0.62–0.90	0.003
(Intercept)	0.18	0.15–0.21	<0.0001	0.17	0.15–0.20	<0.0001	0.18	0.15–0.21	<0.0001	0.18	0.15–0.21	<0.0001
AIC	5440.71			5437.64			5440.80			5442.37		
BIC	5499.08			5502.49			5505.66			5507.23		
Log likelihood	−2711.36			−2708.82			−2710.40			−2711.19		
Number of observations	4844			4844			4844			4844		

AUD = alcohol use disorder (DSM–5); OR = odds ratio; CI = confidence interval; AIC = Akaike information criterion; BIC = Bayesian information criterion; ^a^ measurement from baseline; ^b^ measurement from FU1; ^c^ measurement from FU2; ^d^ reference category: financially autonomous; ^e^ reference category: single.

**Table 3:** Probabilities of alcohol use disorder predicted by the logistic regression (Model 1) across different levels of parental monitoring and active coping.

	Parental Monitoring			
	Low		Median/High	
	P	SE	P	SE
Active coping				
Median/Low	0.35	0.02	0.28	0.01
High	0.29	0.02	0.30	0.01

P = probability; SE = standard error of the probability.

## Data Availability

The data presented in this study are available on request from the corresponding author. The data are not publicly available due to the conditions specified in the data protection contract for this study.
